# Sarcopenia as a Risk Factor for Alzheimer’s Disease: Genetic and Epigenetic Perspectives

**DOI:** 10.3390/genes15050561

**Published:** 2024-04-27

**Authors:** Stuart M. Raleigh, Kayleigh J. A. Orchard

**Affiliations:** 1 Centre for Health and Life Sciences, Coventry University, Coventry CV1 5FB, UK; 2 School of Life, Health and Chemical Sciences, Open University, Milton Keynes MK7 6AA, UK; kayleigh.orchard@open.ac.uk

**Keywords:** Sarcopenia, Alzheimer’s Disease, Genetics, Epigenetics, Non-coding RNAs

## Abstract

Sarcopenia, defined as the age-associated loss of muscle mass and increased fragility with age, is increasing worldwide. The condition often precedes the development of Alzheimer’s disease, thereby decreasing the levels of mobility and physical activity in those affected. Indeed, the loss of muscle mass has, in some studies, been associated with an increased risk of Alzheimer’s disease and other dementias. However, a detailed understanding of the interplay between both conditions is not available and needs to be thoroughly addressed. In the following review, we focus on several genes, specifically *APOE*, *BDNF*, *ACE*, *FTO*, and *FNDC5*, that have been associated with both conditions. We also discuss the epigenetic regulation of each of these genes along with non-coding RNAs (ncRNAs) that may have a role in the development of both the sarcopenic and Alzheimer’s disease phenotypes. Finally, we assert that the application of systems biology will unravel the relationship between sarcopenia and Alzheimer’s disease and believe that the prevention of muscle loss in older age will reduce the incidence of debilitating cognitive decline.

## 1. Background

Sarcopenia, defined as the age-related loss of skeletal muscle and function [1], is known to predispose to an increased risk of falls and progressive loss of mobility with advancing years [2,3]. The condition is typically divided into two sub-categories whereby primary sarcopenia is an age-associated malady with no other evident causes and secondary sarcopenia is linked to causal factors, such as inflammatory disease, organ failure, and reduced physical activity [4,5].

The estimated prevalence of sarcopenia varies considerably and has been shown to range from 1–29% in community-dwelling older adults, 14–33% in elders within long-term care facilities, and 10% in those in an acute care setting [6]. Due to an aging population, the prevalence of sarcopenia is likely to increase as the percentage of the population over the age of 60 increases. For example, the World Health Organization (WHO) has projected that, by 2050, 2.1 billion people will pass their 60th year, inferring that over 200 million people will become sarcopenic in the next 40 years [1].

In addition to an increased fall risk and reduced mobility, evidence suggests that sarcopenia is associated with both Alzheimer’s disease (AD) and cognitive decline [7]. For example, Liu and coworkers recently showed that measures of reduced muscle function, including both lean mass and grip strength, correlate with declines in both cognition and memory. They also showed that lower muscle mass is associated with an increased degree of medial temporal lobe atrophy [8]. Likewise, sarcopenia was recently found to be prevalent in both AD patients and those with Lewy body dementia, where it manifested itself as both reduced muscle strength and slower gait speed [9]. Lower scores for the Mini-Mental State Examination (MMSE) and activities of daily living (ADL) were also evident in AD patients with sarcopenia compared to non-sarcopenic AD patients [10]. Furthermore, findings from a large UK Biobank-based study involving data from more than 190, 000 participants, showed that poor hand grip strength directly associates with reduced neurocognitive health and risk of dementia [11]. The link between sarcopenia and/or muscle mass (or function) and AD is, however, ambiguous in studies where fat-to-muscle ratios (FMRs) have been studied. For example, high whole-body FMRs apparently lower AD risk in men but have no effect on the risk of vascular dementia in both sexes [12]. Interestingly, leg FMRs exert a more pronounced inverse association on all causes of dementia risk in men and women [12]. Such data seem somewhat perplexing, as high FMRs are linked to an increased risk of type 2 diabetes [12], which is an independent risk factor for AD [13]. 

If sarcopenia has an impact on predisposition to AD and the associated dementias, then it is plausible to assume that genetic risk factors for sarcopenia might also exacerbate the risk of AD. In the paragraphs below, we highlight five genes that have been associated with both sarcopenia and AD. We discuss how genetic and epigenetic variability within these genes might modify the risk of both conditions but also spotlight areas where the association is less clear. We also discuss some ncRNAs that appear dysregulated in sarcopenia and AD, the emerging role of network biology, and how this will likely explain overlapping pathways in both conditions. 

## 2. Genetics and Epigenetics

The human *APOE* gene resides on chromosome 19 and encodes a protein of 299 amino acids [14], which acts as a component of circulating lipoproteins and is crucial for the transport of lipids and cholesterol between cells of different organs [15]. The *APOE* gene is polymorphic with three well-studied allelic forms (ε2, ε3, and ε4) that lead to amino acid substitutions at positions 112 and 158. These changes result in three common isoforms of *APOE,* designated as apoE2, apoE3, and apoE4, respectively [16]. The *APOE* ε4 variant is a strong risk factor for both late-stage AD [17] and low-grade inflammation [17]. Interestingly, inflammation is also a risk factor for sarcopenia [18]. Although the exact mechanism whereby the *APOE* ε4 variant predisposes to AD is unclear, recent work suggests a paradoxical process for a neurodegenerative disease whereby the APOE4 isoform increases certain brain signaling pathways and synapse density, presumably by a mechanism involving enhanced precursor protein expression [15]. The *APOE* ε4 variant also predisposes to slower walking speed in individuals with mild cognitive impairments (MCI) [19], and slow walking speed is an underlying feature of sarcopenia [20]. However, and somewhat surprisingly, there is evidence that, in individuals with normal BMI, the *APOE* ε4 variant predisposes to higher lean body mass, and in young individuals certain aspects of the condition appear enhanced in *APOE* ε4 carriers [21]. Collectively, these data do not support a firm role for the *APOE* ε4 variant as predisposing to AD in sarcopenic individuals. However, it is important to mention that the influence of the *APOE* ε4 variant on human phenotypes appears to work through an epigenetic mechanism, as the presence of the ε4 allele changes the DNA methylation pattern of the *APOE* gene [22]. Specifically, the ε4 allele adds an additional CpG site to a 12 bp region that already contains 4 CpG sites. Conversely, the ε2 allele removes a CpG site and opens a 33 bp region free of CpG sites [22]. Furthermore, there appears to be a role for CpG islands within the *APOE* gene to act as signals for protein binding and chromatin remodeling [23], and this might also affect the risk of both sarcopenia and AD with advancing age. Therefore, we suggest that studies into the effect of the ε4 allele on muscle- and/or neurocognitive-related conditions should focus more on its epigenetic influences as opposed to a simple fixation on possession of (or lack of) the ε4 allele itself.

Brain-derived neurotrophic factor (BDNF) is a member of the neurotrophin family, with a complex role in supporting the health and longevity of neurons [24]. BDNF is expressed in various brain regions, particularly the hippocampus [25], along with the prostate [26] and skeletal muscle [27]. BDNF has an important role in long-term memory formation and storage [28], and evidence shows that exercise increases the expression of BDNF. For example, intense resistance exercise in both sexes has been shown to increase circulating BDNF levels immediately and 30 min after the intervention [29]. Furthermore, Lira and colleagues [30] found that lower-body resistance exercise was associated with higher BDNF levels compared to full- and upper-body exercise. However, some investigations have not found an increase in BDNF following resistance exercise [31]. For aerobic exercise, even in older-age subjects (65–74 years), significant increases in BDNF have been observed following 12 weeks of treadmill walking [32] and low-intensity prolonged cycling also upregulates BDNF [33]. 

Interestingly, a link between lower levels of BDNF and sarcopenia is evident in the literature. For example, Roh and co-workers [34] showed that reduced plasma BDNF levels were associated with both sarcopenia and dementia in a cohort of community-dwelling adults aged 70–84. Likewise, in sarcopenic patients with Parkinson’s disease [35] or comorbid with respiratory dysfunction, lower levels of BDNF are reported [36]. A recent meta-analysis showed that serum BDNF was lower in those with Alzheimer’s disease compared to healthy controls [37]. However, in peripheral blood samples, Kim and colleagues found that BDNF was lower in AD patients with the lowest MMSE scores, but in early AD, BDNF was higher than in controls [38]. 

Although, in general, it seems that lower BDNF is associated with both sarcopenia and AD, it should be noted that expression of BDNF is modified by the presence of the rs6265 (G196A or Val66Met) variant within exon XI of the gene [39]. The rs6265 variant is known to influence several downstream functions of BDNF, such as hippocampal function and episodic memory [39]. Furthermore, the presence of the A allele removes a CpG binding site within the gene that impacts expression [39]. Importantly, the extent of DNA methylation within promoter regions of the *BDNF* gene has been shown to impact cognitive performance in individuals with major depressive disorder [40]. The role of *BDNF* methylation in the development of AD has also been demonstrated by the work of Xie and co-workers. These authors conducted a 5-year longitudinal study on epigenetic changes in patients with mild cognitive impairment who converted to AD. They measured CpG site methylation at four locations within two promoter regions of the *BDNF* gene and found that elevated methylation of a site within the promoter IV area significantly (HR = 3.51, *p* = 0.013) associated with the conversion of MCI to AD [41].

The Angiotensin-converting enzyme (*ACE*) gene is found on chromosome 17q23 and consists of 26 exons with 25 introns. The gene itself contains at least 160 variant sites and encodes two isoforms with an important role in regulating blood pressure. Specifically, ACE converts angiotensin I to angiotensin II, and this has a vasoconstrictive effect on blood vessels and, therefore, reverses the effect of low blood pressure [42]. The common *ACE* I/D variant that resides within intron 17 of the gene has been associated with a broad spectrum of phenotypes and has long been considered an important factor related to both strength and endurance [43]. However, although the I/D variant has been associated with sarcopenia, the direction of association is still unclear. For example, the II genotype has been found to predispose to sarcopenia in a Brazilian population [44] but in elderly Indonesians, it was the DD genotype that was associated with the same condition [45]. Furthermore, in women, the DD genotype seems to protect from sarcopenic obesity [46]. As with sarcopenia, the role of the *ACE* I/D variant as a risk factor for AD is somewhat opaque, but a recent meta-analysis, using data from 65 studies, found some evidence to suggest that the I allele increased risk [47]. This finding is consistent with an older, large-scale study that also found that the I allele increased the risk of AD across diverse populations [48]. Interestingly, and perhaps inconsistent with the role of the I allele as a risk factor for AD, *ACE* D allele carriers with mild cognitive impairments (MCI) perform worse on auditory–verbal learning tests compared to those without a D allele [49]. A similar association between D allele carriage and recall in MCI patients was also found by Zhang and colleagues [50]. 

It is important to recognize that the regulation of the *ACE* gene is under strong epigenetic control via DNA methylation of CpG sites within its promoter. ACE expression is also controlled by histone modifications [51]. Indeed, the methylation status of the *ACE* gene has an indirect and possibly profound effect on the stress response and predisposition to depression, as it can modulate the levels of cortisol [52]. Elevated cortisol has been linked to both sarcopenia [53] and AD [54]. Hence it seems that the methylation status of the *ACE* gene may be more important than certain sequence variants in influencing a range of physiological phenotypes [55]. 

The *FTO* (fat mass and obesity-associated protein) gene resides on chromosome 16 in humans and encodes a non-heme dioxygenase Fe(II) and 2-oxoglutarate-dependent dioxygenase [56], which functions as a mRNA demethylase [57]. In recent years, variation within the *FTO* gene has been studied in a wide variety of phenotypes related to weight gain [58,59] cardiovascular fitness [60], and elite sporting ability [61]. Shu Ran and colleagues (2020) used a genome-wide association (GWAS) approach in a large Caucasian cohort to identify 29 single nucleotide polymorphisms (SNPs) associated with sarcopenia [62]. Furthermore, the role of *FTO* SNPs in sarcopenia has also been demonstrated in COPD patients by Attaway and coworkers using a GWAS design followed up with in vitro FTO knockdown experiments. In their studies, they found that *FTO* knockdown in mice yielded a sarcopenic profile that was also exacerbated by hypoxia [63]. Interestingly, Khanal and colleagues (2020) have shown that the *FTO* rs9939609 variant seems to be associated with sarcopenia, but the association was dependent on the definition used to describe the condition. Specifically, the variant modified the risk of sarcopenia when defined by both the percentage of muscle mass and the skeletal muscle mass index, but the association disappeared when using a definition for sarcopenia based on the European Working Group on Sarcopenia in Older People (EWGSOP) [64]. This clearly shows the necessity of using a standardized set of criteria for disease definition across all studies. 

Variation within the *FTO* gene seems also to impact on the risk of AD. Using a cohort of nearly 2000 Caucasian cases and controls, the rs6499640 SNP within intron 1 of the gene was found to significantly associate with AD, while the rs10852521, rs16945088, and rs8044769 SNPs approached a significant p value for association [65]. The same authors also found that in Hispanics, the rs17219084, rs11075996, and rs11075997 SNPs were significantly associated with the disease. Likewise, a significant association between the *FTO* rs9939609 variant (AA genotype) and both AD and dementia was demonstrated by Keller and colleagues as part of the Kungsholmen project [66]. Furthermore, these authors also discovered an interaction effect between the *FTO* AA and *APOE* ϵ4 genotypes that increased the risk of both AD and dementia independent of other potentially confounding factors, such as diabetes, physical activity, and BMI [66]. 

It seems then that variation within the *FTO* gene can modify the risk of both sarcopenia and AD. However, although most studies have focused on population-based cohort studies, the effect of epigenetic modification of the *FTO* gene and the risk of both conditions has largely been ignored. Interestingly, the *FTO* gene is regulated at the epigenetic level by increased CpG site methylation/demethylation within the promoter by such factors as smoking, dietary triglycerides, curcumin, and Vitamin B12 [67]. Furthermore, promoter *FTO* methylation levels were shown to inversely correlate with birth weight in a Chinese population [68]. Indeed, a detailed understanding of how *FTO*-promoter methylation impacts on lean muscle mass and risk of AD, as a function of age, would prove useful.

Although a detailed commentary on the myokines is beyond the scope of this paper, we assert that these molecules and their aberrant expression may have a significant role to play in the development of both sarcopenia and AD. The myokines, which consist of a large group of small cytokine-based peptides, are secreted by muscle cells during the process of muscle contraction [69]. Indeed, this phenomenon is one way in which muscles communicate with other tissues of the body and points also towards an endocrine role for muscle that perhaps has previously been overlooked [69]. Irisin, formally discovered in 2012 [70], is a myokine that activates a range of signaling pathways in a variety of different tissues, including the mitogen-activated protein kinase (MAPK) pathway in neuronal tissue, the ERK1/2 pathways in cardiac microvascular endothelial cells [71], and the activation of telomerase in hepatocytes [72]. Circulating irisin levels have been shown to be lower in sarcopenic patients [73,74], and resistance training upregulates irisin via the AMPK/PGC-1α/irisin signaling pathway [75]. There is now growing interest in the role of Irisin in AD as the molecule has been shown to potentially protect from the deposition of amyloid-β (Aβ) protein in the brain, a key molecular signature of AD pathology, by upregulating astrocytic release of Aβ-degrading enzymes [76]. Indeed, this may be a mechanism whereby exercise protects from the risk of AD [76]. Furthermore, elevated irisin has been associated with improved global cognition and memory in adults at risk of dementia following ten weeks of physical or cognitive training [77]. 

**Figure 1:**
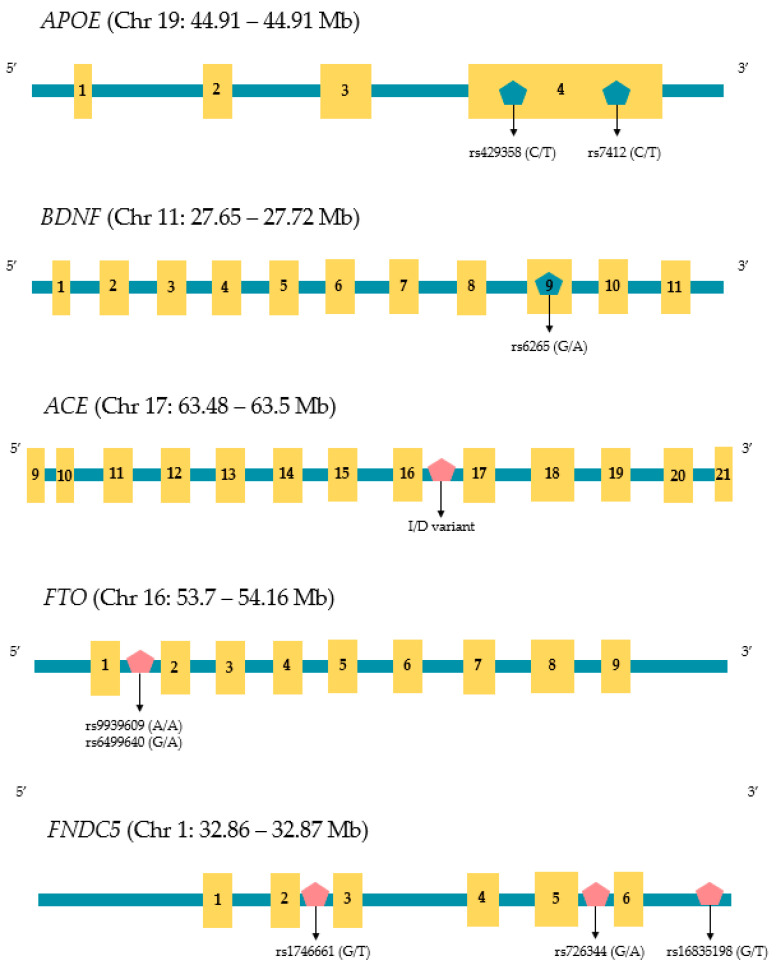
Chromosomal locations and approximate sizes of the genes reviewed in this article. Horizontal bars show intronic regions and the yellow boxes represent exons which are numbered according to their position. Relevant single nucleotide polymorphisms (SNPs) and the ACE indel are also shown along with their relative positions. Gene structure and information on each SNP were obtained by reference to the University of California Santa Cruz Genome Browser (hg38) at: https://genome.ucsc.edu/ (accessed on 24 April 2024).

In humans, irisin is encoded by the *FNDC5* (Fibronectin type III Domain Containing 5) gene that is found on chromosome 1. There are three variants of FNDC5 distinguished by sequences that code for different signal peptides and C-terminal domain regions [78] Variation within the *FNDC5* promoter has been associated with a variety of phenotypic changes. For example, Lima Filho and colleagues reported that the rs1746661 variant (T allele) influences glucose metabolism within the brains of elderly individuals, which affects the risk of AD [79]. Using a mouse model, Madhu and coworkers have shown that *FNDC5* knockout reduces the benefits of exercise-induced cognitive enhancement, which supports the role of irisin in protecting from AD [80]. Furthermore, two polymorphisms within the *FNDC5* promoter (rs16835198 and rs726344) are both known to impact insulin sensitivity [81], and insulin sensitivity is a contributing factor for developing sarcopenia [82,83]. In addition to *FNDC5* sequence variation, the gene is controlled epigenetically by a large CpG site, located at −52~−442 bp within two of the *FNDC5* genes (variant 2 and 3), and methylation of this region has a significant effect on the expression of the gene. [78]. However, to our knowledge, a detailed understanding of the methylation status of this CpG site and its influence on both sarcopenia and AD risk has yet to be established. The structure, size, and location of relevant SNPs discussed in this review are shown in Figure 1. 

Noncoding RNAs (ncRNAs), such as microRNAs (miRNAs) and long noncoding RNAs (lncRNAs), do not code for proteins but regulate gene expression through mRNA binding and transcriptional silencing, in the case of miRNAs [84]. In addition to mRNA binding, lncRNAs also regulate the overall process of gene expression through multiple regulatory processes, including the regulation of both histone modification and DNA methylation [85]. Lnc RNAs also act as protein scaffolds that can bring together multiple proteins to enhance biological functions [85]. There are multiple studies that show associations between ncRNAs and sarcopenia. For example, Faraldi and coworkers recently reported the downregulation of hsa-miR-221-3p, hsa-miR-374b-5p, hsa-miR-146a-5p, hsa-miR-126-5p, and hsa-miR-425-5p and the upregulation of hsa-miR-145-5p and hsa-miR-25-3p in post-menopausal women with muscle wasting [86]. Sarcopenic patients have also been shown to have downregulated levels of miR-133a, miR-133b, miR-206, miR-208b, and miR-499 [87]. Indeed, the role of ncRNAs in sarcopenia is further strengthened by the observation that certain ncRNAs, such as miR-155-5p, miR-421-3p, miR-425-5p, and miR-495-3p, appear repressed in those with the condition, but upon appropriate exercise intervention, expression levels return to normal [88]. Altered expression of ncRNAs maybe useful for the diagnosis and management of sarcopenia. For instance, the levels of circulating miR-1-3p, which can activate components of the Akt/mTOR pathway, are elevated in sarcopenic patients with heart disease and may be useful as a biomarker for the prediction of sarcopenia in these individuals [89]. Likewise, Valášková and colleagues [86] have shown that a profile of multiple ncRNAs, including miRNA-1, miRNA-29a and miRNA-29b, are increased in older individuals (aged 55-80 years) with poor muscle performance, whereas they detected decreased expression of miRNA-206, miRNA-133a, miRNA-133b, miRNA-208b, and miRNA-499 correlated with the same phenotype [86]. Indeed, the authors speculated that their data may be useful in the development of miRNA biomarkers for sarcopenia. 

It seems likely then that ncRNAs may have a role to play in sarcopenia. However, it is more difficult to ascertain whether these molecules have a causative role in the condition or whether they are simply an artifact of muscle atrophy or other degenerative process during the aging process. Furthermore, is it possible that changes in ncRNAs that might predispose to sarcopenia may also increase the risk of AD? Indeed, many of the ncRNAs mentioned above (that have a role in sarcopenia) are also associated with AD. For example, the levels of hsa-miR-126-5p were shown to be altered in neutrophil-derived microvesicles of AD patients compared to controls, leading to possible blood–brain barrier dysfunctions that may predispose to AD [90]. Yuan and colleagues used mechanistic studies to establish that upregulation of hsa-miR-425-5p promotes tau phosphorylation and apoptosis in AD [91]. Indeed, tau hyperphosphorylation is a hallmark of AD pathology [92]. Interestingly, upregulation of has-miR-145p was recently shown to be a predictor of both AD and mild cognitive impairment (MCI) in a small cohort of patients. The same study found that hsa-miR-145 also improved the predicted performance of AD patients on the Mini-Mental State Examination (MMSE) [93]. Furthermore, both hsa-miR-29a and hsa-miR-29b have been shown to be altered in AD cohorts. Specifically, Peña-Bautista and coworkers [94] found upregulated levels of hsa-miR-29a that, in combination with several other ncRNAs, tentatively associated with AD. However, hsa-miR-29a was found to be downregulated in Egyptian patients with AD [95]. Studies into the interaction of lncRNAs with miRNAs have shown that hsa-miR-155-5p may play a role in neuron projection development and neuron morphogenesis in AD [96]. Furthermore, the authors infer that hsa-miR-155-5p may have potential as a clinical biomarker for AD [96]. As with sarcopenia, we must also be cautious in inferring direct causative roles for ncRNAs in AD. Furthermore, although we have highlighted overlaps between ncRNAs that may appear dysregulated in both conditions, definitive mechanisms for how these molecules enhance the risk of both are not yet understood. It is important to remember that ncRNAs, for example, miRNAs, often have multiple target mRNAs that they can regulate, and it is possible that some observations of their altered expression in disease processes (like sarcopenia and AD) may be coincidental findings or due to confounding factors within patient cohorts. A summary of risk genes, non-coding RNAs, and microRNAs discussed in this review is shown in Table 1. 

In the above paragraphs, we have limited discussion to how variability (both genetic and epigenetic) within five genes (*APOE*, *BDNF, ACE*, *FNDC5,* and *FTO*) might impact on both sarcopenia and AD risk. We have also included a commentary on the role of ncRNAs that overlap in both conditions. However, these are complex conditions and unlikely to be solely due to changes within a small number of genes per se. To support this view, it has now been demonstrated that 270 differentially methylated regions of the genome exist between patients with AD compared to controls. Furthermore, the methylation changes were shown to profoundly impact protein networks and transcriptomic, proteomic, and chromatin accessibility of specific regions of the brain in AD patients [97]. However, overlapping epigenetic and interatomic changes between AD and sarcopenia are only just starting to emerge. For example, it was recently shown, using protein-interaction networks, that the dysregulated expression of five overlapping mitochondrial hub genes (*NDUFAB1*, *UQCRC1*, *UQCRFS1*, *NDUFS3*, and *MRPL15*) and associated networks may be important in both musculoskeletal aging (sarcopenia) and AD [98]. Indeed, it is the authors’ assertion that a systems-biology-based approach to identify common aspects, and potentially new biomarkers for both conditions, should now be a priority. This approach has been adopted by Yedigaran and colleagues where divergent and shared miRNA regulatory pathways in both cachexia and sarcopenia were identified [99] but extending this work to overlapping miRNA networks between sarcopenia and AD pathways, especially in relation to physical activity, might also prove beneficial. Finally, when it comes to both sarcopenia and AD, we are faced with a possible cause-and-effect question. Specifically, does sarcopenia, which is postulated to be due, at least in part, to systemic inflammation [100], promote a cascade of events that leads to AD, or does AD lead to sarcopenia purely by a reduction in physical activity, and subsequent loss of muscle mass, in those affected? Whichever of these two scenarios is at play, an integrated genetic, epigenetic, and systems-biology-based signature would help to identify at-risk individuals and allow for personalized interventions.

## Figures and Tables

**Table 1:** Summary of the role of risk genes, non-coding RNAs, and microRNAs discussed in this review with relevance to both sarcopenia and Alzheimer’s disease.

Risk Genes, Non-Coding RNAs, and MicroRNAs	Potential Epigenetic Regulation	Sarcopenia	Alzheimer’s Disease
*APOE* ε4 variant	DNA methylation	Inflammation is a risk factor for sarcopenia, and the *APOE* ε4 variant is associated with low-grade inflammation [17].	*APOE* ε4 variant is a strong risk factor, associated with amyloid deposition increase and an earlier stage of onset [17].
*BDNF*	DNA methylation	Reduced plasma BDNF associated with sarcopenia and dementia in adults aged 70–84 [34]. Lower levels of BDNF reported in patients with Parkinson’s [35].	Serum BDNF lower in patients with Alzheimer’s compared to healthy controls [37]. BDNF lower in peripheral blood samples of patients with the lowest MMSE sores but higher in early Alzheimer’s disease [38]. In patients with mild cognitive impairment (MCI) that progressed to Alzheimer’s disease; elevated methylation of a CpG site within the promoter of *BDNF* was significantly associated with the conversion of MCI to Alzheimer’s [41].
*ACE* I/D variant	DNA methylation Histone Modifications	In a Brazilian population, the II genotype of the *ACE* I/D variant predisposes to sarcopenia [44]. In an elderly Indonesian population, the DD genotype was found to be associated with sarcopenia [45].	Meta-analysis suggests that the I allele of the *ACE* I/D variant increased the risk of Alzheimer’s Disease, which is supported by Lehmann and colleagues [47,48].
		Methylation of *ACE* has an indirect effect on the stress response and predisposition to depression, as it can modulate the levels of cortisol, and elevated levels of cortisol have been associated with sarcopenia and Alzheimer’s Disease [53,54].	
*FTO*	DNA methylation	GWAS identified 29 SNPs in a Caucasian cohort that were associated with sarcopenia [61]. GWAS followed by *FTO* knockdown in mice produced a sarcopenic profile [63]. The *FTO* rs9939609 variant is associated with sarcopenia when defining sarcopenia using percentage of muscle mass and skeletal muscle mass index but not when using the EWGSOP definition of sarcopenia [64].	The rs649960 SNP within intron 1 of *FTO* significantly associates with Alzheimer’s in a large Caucasian cohort, and rs10852521, rs16945088, and rs8044769 were close to significance [65]. In a Hispanic population, rs17219084, rs11075996, and rs11075997 are significantly associated with Alzheimer’s disease [65]. Significant association found between the rs9939609 variant of *FTO* (AA genotype) and Alzheimer’s disease and dementia [66].
*FNDC5* (Irisin)	DNA methylation	Circulating Irisin levels are lower in patients with sarcopenia [73,74]. The rs16835198 and rs726344 variants of *FNDC5* can impact insulin sensitivity, which is a contributing factor to the development of sarcopenia [82,83].	The rs1746661 variant of *FNDC5* influences glucose metabolism in elderly individuals which affects the risk of Alzheimer’s disease [79]. *FNDC5* knockout in a mice model reduces the benefits of exercise-induced cognitive enhancement, which supports the role of Irisin in Alzheimer’s disease protection [80].
hsa-miR-221-3p	Non-coding RNA	Downregulated in post-menopausal women with muscle wasting [86].	N/A
hsa-miR-374b-5p	Non-coding RNA		N/A
hsa-miR-146a-5p	Non-coding RNA		N/A
hsa-miR-126-5p	Non-coding RNA		Altered levels in neutrophil-derived microvesicles of patients with Alzheimer’s disease [90].
hsa-miR-425-5p	Non-coding RNA		Upregulation promotes tau phosphorylation, which is a hallmark of Alzheimer’s disease pathology [92], and apoptosis in Alzheimer’s disease [91]. Upregulation also shown to be a predictor of both Alzheimer’s disease and mild cognitive impairment [93].
hsa-miR-145-5p	Non-coding RNA	Upregulated in post-menopausal women with muscle wasting [86].	N/A
hsa-miR-25-3p	Non-coding RNA		N/A
miR-133a	Non-coding RNA	Downregulated in patients with sarcopenia [87].	N/A
miR-133b	Non-coding RNA		N/A
miR-206	Non-coding RNA		N/A
miR-208b	Non-coding RNA		N/A
miR-499	Non-coding RNA		N/A
miR-155-5p	Non-coding RNA	Repressed in sarcopenia, but expression levels return to normal upon exercise intervention [88].	May play a role in neuron projection development and neuron morphogenesis in Alzheimer’s disease and could also act as a clinical biomarker for Alzheimer’s disease [96].
miR-421-3p	Non-coding RNA		N/A
miR-495-3p	Non-coding RNA		N/A
miR-495-5p	Non-coding RNA		N/A
miR-1-3p	Non-coding RNA	Elevated expression in patients with sarcopenia and heart disease may be beneficial to use as a biomarker to predict sarcopenia in patients with heart disease [89].	N/A
miRNA-1	Non-coding RNA	Increased expression in patients aged 55 to 80 with poor muscle performance [87].	N/A
miRNA-29a	Non-coding RNA		Upregulated levels tentatively associated with Alzheimer’s disease [94].
miR-29b	Non-coding RNA		Downregulated in Egyptian patients with Alzheimer’s disease [95].
miRNA-206	Non-coding RNA	Decreased expression in patients aged 55 to 80 with poor muscle performance [87].	N/A
miRNA-133a	Non-coding RNA		N/A
miRNA-133b	Non-coding RNA		N/A
miRNA-208b	Non-coding RNA		N/A
miRNA-499	Non-coding RNA		N/A
